# Differential IL-18 Dependence of Canonical and Adaptive NK Cells for Antibody Dependent Responses to *P. falciparum*

**DOI:** 10.3389/fimmu.2020.00533

**Published:** 2020-03-31

**Authors:** Samuel Sherratt, Avnish Patel, David A. Baker, Eleanor M. Riley, Martin R. Goodier

**Affiliations:** ^1^Department of Infection Biology, London School of Hygiene and Tropical Medicine, London, United Kingdom; ^2^School of Biological Sciences, Institute of Immunology and Infection Research, University of Edinburgh, Edinburgh, United Kingdom

**Keywords:** NK cells, *Plasmodium falciparum*, IL-18, FcεR1γ, CD57

## Abstract

Human adaptive natural killer (NK) cells have diminished reliance on accessory cytokines for their activation whilst being efficiently activated by infected host cells in conjunction with pathogen specific antibodies. Here, we show that potent antibody-dependent NK cell responses are induced by *Plasmodium falciparum* infected erythrocytes (iRBC) in peripheral blood mononuclear cells (PBMC) from malaria-exposed Gambian individuals in the presence of autologous sera, which are absent in those from malaria-naïve UK individuals. However, malaria hyper-immune serum promotes rapid NK cell responses to iRBC in cells from both Gambian and UK individuals. Among Gambians, highly differentiated, adaptive (CD56dimFcεR1γ-CD57+) NK cells dominate both antibody-dependent NK cell IFN-γ responses and degranulation responses, whereas among UK individuals these responses are predominantly found within canonical, highly differentiated CD56dimFcεR1γ+CD57+ NK cells. Indeed, overall frequencies of adaptive, FcεR1γ-CD57+ NK cells are significantly higher among Gambian donors compared to HCMV-infected and HCMV-uninfected UK adults. Among UK individuals, antibody-dependent NK cell IFN-γ responses to iRBC were dependent on IL-18 whereas among Gambians, the predominant adaptive FcεR1γ- NK cell response was IL-18 (and accessory cell) independent (although the lower frequency response of canonical FcεR1γ NK cells did rely on this cytokine).

## Introduction

Natural killer cells integrate signals from both inflammatory mediators and acquired T and B cell responses, enabling them to bridge innate and adaptive immunity, however, the intrinsic differentiation state of these cells determines which combinations of signals contribute to their activation and function (1). In human peripheral blood CD56bright NK cells are a minor population and considered as the least differentiated subset. These cells respond predominately to innate cytokines and T cell-derived interleukin-2 (IL-2) via constitutive expression of receptors for IL-12 and IL-18, together with the high affinity CD25/CD122 complex for IL-2 (1). Only rare populations of CD56bright cells respond to antibody-dependent signals, and only in some individuals. In contrast, circulating CD56dim cells almost universally express CD16 (FcγRIIIA) and have reduced constitutive expression of cytokine receptors (1). There is, however, considerable additional diversity within CD56dim NK cells: progressive acquisition of CD57, a cell surface moiety expressed with increasing differentiation, is associated with higher expression of CD16 and further reductions in cytokine receptor expression and cytokine responsiveness (2, 3). Human cytomegalovirus infection is strongly associated with further diversification of the natural killer cell repertoire (4–7) including expansion of populations of CD57+NK cells bearing the c-type lectin-like receptor, NKG2C. Ultimately, this subset is highly represented amongst adaptive NK cells, which have lost expression of the proteomyeloid zinc finger (PLZF) molecule and the membrane adaptor protein FcεR1γ, switching to usage of components of the PI3 kinase signaling cascade including CD3ζ and ZAP70 and which can mount antibody dependent responses independently of cytokines (6, 7). Indeed the expression of transcripts for IL-12 and IL-18 receptors and associated signaling components is lost in adaptive NK cells (7).

Acquisition of non-sterilizing immunity to malaria is characterized by a progressive reduction of inflammation with the gradual development of acquired cellular and humoral responses (8, 9). Immune responses to erythrocytic stages of the parasite lifecycle are associated with naturally acquired immunity (10). Pathogen- associated molecular patterns from blood stage *P. falciparum* parasites, either alone or in association with haemozoin (the residue of hemoglobin digestion by the parasite), are potent inducers of NK cell activating cytokines, including IL-12, IL-18, and type 1 interferons (11–13). Inflammatory cytokines are associated both with control of blood stage parasitaemia and with onset of malaria pathology (14–19) but tend to diminish in concentration with increasing levels of exposure and clinical immunity. On the other hand, malaria-specific IgG1 and IgG3 antibodies with the potential to induce antibody dependent cellular cytotoxicity (ADCC) have long been described to be potentially protective against malaria (20, 21).

Natural killer cells are rapidly activated during controlled human malaria infections of malaria non-immune individuals and *in vitro* by *P. falciparum* infected erythrocytes (22–26). A common feature of all of these models is contact-dependent and cytokine-dependent activation of less differentiated NK cells (CD56bright or CD56dimNKG2A+KIR–) by myeloid accessory cells (22, 23, 27, 28). More recently, the role of antibody in targeting *P. falciparum* merozoites and iRBC for growth inhibition and killing by human natural killer cells has been demonstrated, with significant contributions of responses to the variable, erythrocyte surface-expressed malaria antigens, PfEMP1 and RIFIN (29). Moreover, frequencies of FcεR1γ- adaptive NK cells correlated with lower parasitaemia and resistance to malaria in prospective studies in Mali and increasing frequencies of PLZF- adaptive NK cells were associated with longer term resistance (30).

In addition to their efficient lysis of iRBCs and inhibition of parasite growth, adaptive NK cells may also contribute to effective antimalarial immunity due to their reduced reliance on inflammatory cytokines, which contribute to malarial disease. Our previous studies have shown that NK cell differentiation proceeds rapidly in early life in a Gambian population, with near-maximal frequencies of CD57+NKG2C+ cells observed by the age of 10 years and coincident loss of NK cell responsiveness to exogenous IL-12 and IL-18 (31). We hypothesized that *in vitro* responses of less differentiated NK cells to *P. falciparum*-infected erythrocytes would be strongly dependent on accessory cytokines for their activation, whilst more differentiated subsets would progressively lose this dependence with adaptive NK cells making potent antibody-dependent but cytokine-independent responses. We therefore compared NK cell differentiation phenotype and *in vitro* responses to *P. falciparum*-infected erythrocytes in Gambian and European individuals in the presence or absence of autologous serum antibody.

We observed high frequencies of FcεR1γ- adaptive NK cells and potent antibody-mediated anti-malarial NK cell responses in Gambian individuals compared to European donors. However, NK cell activation with iRBC in the presence of a standard malaria hyper-immune serum stimulated potent NK cell degranulation and IFN-γ production in both Gambian and UK individuals. In UK individuals, responses were strongest in CD56dimCD57+ NK cells, and required endogenously produced IL-18 but not IL-12. In contrast, in Gambian adults, adaptive NK cells dominated antibody-dependent degranulation and IFN-γ responses and were IL-18 independent. These data demonstrate for the first time that IL-18 synergises for antibody-dependent activation of canonical FcεR1γ+ NK cells by *P. falciparum* but that subsequent maturation of NK cells to the adaptive FcεR1γ- state has the potential to uncouple these responses from the inflammatory cascade, thereby facilitating clearance of iRBC without the need for induction of pathogenic inflammatory cytokines.

## Materials and Methods

### Study Subjects

Heparinized blood samples were collected from 26 healthy Gambian adults (13 male; 13 female, mean age ± SD = 30.3 ± 8.7) as part of a previously published field study (31), and from 15 HCMV seropositive (7 male; 8 female, mean age ± SD = 36.3 ± 10.3) and 17 HCMV seronegative (7 male; 11 female, mean age ± SD = 38.0 ± 10.3) healthy UK volunteers. Samples were centrifuged and separated into plasma and peripheral blood mononuclear cells (PBMC) and cryopreserved. Exclusion criteria included known chronic disease, confirmed infections during the previous 3 months or an axillary temperature of ≥38°C. All Gambian subjects were shown to be negative for malaria infections by thick blood smear. These studies were approved by the London School of Hygiene and Tropical Medicine Research Ethics Committee and the Gambia Government/MRC Gambia Joint Ethics Committees (references 6034, 6237, and SCC1449v2).

### Cell Preparation

PBMC were isolated by standard ficoll hypaque centrifugation and cryopreserved in 5% DMSO in fetal calf serum at −80°C overnight in Mr. Frosty™ freezing containers prior to transfer into liquid nitrogen. Cells were recovered by thawing rapidly into culture medium and repeated washing, and were rested at room temperature for 3 h prior to stimulation. Viability of recovered cells was >95% and yields ranged from 30 to 65%. NK cells were enriched from PBMC of 6 Gambian individuals using an NK cell purification kit in accordance with manufacturers protocols (Miltenyi Biotech, UK). NK cell purities were (mean ± sd) 88% ± 6% of viable lymphocytes.

### Plasma Samples and Serological Assays

HCMV serostatus of Gambian and UK study subjects was confirmed using a commercially available ELISA to detect anti-HCMV IgG (Demeditec, Germany). All 26 Gambian individuals studied were confirmed to be HCMV seropositive and were also found to have prior exposure to *Plasmodium falciparum* malaria as demonstrated by seropositivity for IgG antibodies to *P. falciparum* apical merozoite antigen 1 (AMA-1) using a validated in house ELISA assay. Samples from HCMV- and HCMV+ UK were selected to reflect a similar distribution of age and sex to Gambian samples.

### Parasite Cultures and Parasite Enrichment

Uninfected erythrocytes (uRBC) were obtained from the National Blood Service, London, UK on a weekly basis and stored at 4°C. Erythrocytes infected with *P. falciparum* strain NF54 were routinely synchronized and cultured at 5% haematocrit, 0.5–2.5% parasitaemia in 75 ml filtered top flasks (Nunc) according to published protocols (32) and were tested regularly for *Mycoplasma spp*. contamination using LookOut Mycoplasma QPCR Detection Kit (Sigma-Aldrich, UK). Parasitaemia was monitored by microscopic examination of methanol-fixed, Giemsa-stained blood films. Late-stage schizonts were harvested from cultures of 10–12% parasitaemia via centrifugation over 65% warmed Percoll solution (2,500 g for 10 min without brake), were washed twice in culture medium to remove any traces of Percoll These were then cultured by mixing with fresh red blood cells in culture medium and letting parasites invade for 4–5 h followed by subsequent treatment with 5% sorbitol to remove parasites that had not re-invaded. Synchronous cultures (5–10% parasitaemia) were then grown for a further 38 h and trophozoites and early schizont stages harvested by percoll purification.

### *In vitro* Stimulation of NK Cells

PBMC or purified NK cells were cultured with uninfected (uRBC) or *P. falciparum*-infected erythrocytes (iRBC) at a ratio of 3 RBCs per leucocyte. Typically 4 × 10^5^ PBMC or purified NK cells were used for each assay point.

For antibody-dependent assays, autologous plasma or a WHO standard anti-malarial serum (National Institute for Biological Standards and Control (NIBSC), UK; (33) was added to the cultures at a final concentration of 1% (v/v) and the final total serum concentration normalized to 5% in with FCS. Negative control cultures contained FCS alone or antibody-depleted autologous or anti-malarial serum (depleted of IgG using a HiTrap® protein G column, Merck, Sigma, UK). Overall capacity for antibody-dependent NK cell activation was assessed for each PBMC or NK cell sample using 100 ng/ml Rituximab (Rituxan®, Genetech, USA) and Raji B cells as targets (NIBSC, UK) at a PBMC: target cell ratio of 5:1. For functional cytokine blockade, a preservative-free, low endotoxin formulation of anti-IL-12 (mIgG1, BD Biosciences, UK), anti-IL-18 (mIgG1, BD Biosciences, UK) or isotype matched control antibody were added at a final concentration of 3 μg/ml for 30 min prior to stimulation. Cells were cultured for a total of 6 h. To facilitate intracellular IFN-γ staining or CD107a accumulation, Brefeldin A and Monensin (Both from BD Bioscience, UK) were added after 1 h of culture at final concentrations of 1:1000 and 1:1500, respectively.

### Flow Cytometric Analysis of NK Cells

*Ex vivo* or *in vitro* analysis of NK cell subsets was performed using the following reagents: anti-CD3-V500 (clone UCHT1) (BD Biosciences), anti-CD56-BV605 (clone HCD56), anti-IFN-γ-BV785 (clone 4S.B3), anti-CD16-PeDazzle-594 (clone 3G8) (all from Biolegend, London, U.K.); anti-CD16-APC (clone CB16), anti-CD25-PerCPCy5.5 (clone BC96), anti-CD57-e450 (clone TB01), CD218-PE (anti-IL-18Rα) (clone H44) (all from Thermo-Fisher) anti-NKG2C-PE (clone 134591) (R&D systems). Rabbit polyclonal IgG anti-FcεR1γ FITC was obtained from Millipore, UK. Anti-CD107a-e660 (clone 1D4B) (Biolegend) was added to the culture at 2 μl per 100 μl for the whole culture period.

For staining of cell surface markers, cells were harvested into 96 well round-bottom tissue culture plates, washed in FACS buffer (PBS, 0.5% FCS, 0.05% sodium azide, 2 mM EDTA) and Fc receptors blocked for 5 min with Fc Receptor (FcR) Blocking Reagent (Miltenyi Biotec). Reagents for surface markers, supplemented with a fixable viability dye (Fixable Viability Stain 700; BD Biosciences) were added to the cells for 30 min. Cells were washed in FACS buffer, fixed and permeabilized according to the manufacturer's instructions using Cytofix/Cytoperm Kit (BD Biosciences) or Foxp3/Transcription Factor Fixation/ Permeabilisation Kit (eBiosciences) and then stained for intracellular markers with repeated FcR blockade for 20 min. Finally, cells were washed and resuspended in FACS buffer before acquisition on a BD LSRII flow cytometer using BD FACSDiva software. Data were analyzed using FlowJo V10 (Tree Star, Oregon, U.S.A). Flow cytometry gating was established using FMO controls or unstimulated cells. Between 1,000 and 10,000 gated total NK cell events were acquired, equivalent to 790–9560 CD56dim cell events. For *ex-vivo* analysis, samples with fewer than 100 events in each phenotyped NK cell subset were excluded. For *in vitro* stimulations with iRBC+ immune serum, a mean of 1940 CD107a+ and 320 IFN-γ+ CD56dim+ NK cells were acquired.

### Statistical Analysis

Statistical analysis was performed using GraphPad Prism version 7.04 (GraphPad, California, U.S.A.). Intergroup comparisons were made using Mann-Whitney U test and paired comparisons between culture conditions or cell subsets were made using Wilcoxon signed-rank test. Correlations were performed using Spearman's correlation analysis. Significance levels are assigned as *p < 0.05, **p < 0.01, ***p < 0.001, and ****p < 0.0001 for all tests.

## Results

Our previous studies have demonstrated rapid differentiation of peripheral blood NK cells in Gambian children with frequencies and geometric mean fluorescence intensity (MFI) for the late differentiation marker CD57 stabilizing to levels reached in adult Gambians by the age of 14 years (31). With progressive differentiation and adaptation, human NK cells demonstrate reduced responsiveness to cytokines accompanied by enhanced antibody-dependent activation (6, 7). We therefore hypothesized that highly differentiated and adaptive NK cells would demonstrate superior antibody dependent responses to *P. falciparum* infected erythrocytes.

### Antibody-Dependent Responses of Gambian and UK Individuals

**Figure 1:**
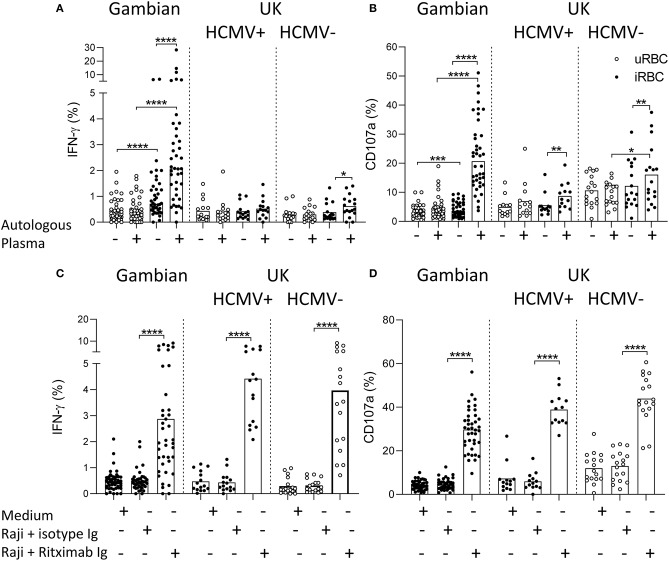
Antibody-dependent NK cell responses to *Plasmodium* infected erythrocytes. **(A,C)** IFN-γ and **(B,D)** CD107a responses measured within gated CD56dim NK cells of Gambian (*n* = 26) and HCMV+ (*n* = 15) and HCMV– (*n* = 17) UK individuals. **(A,B)** cells cultured either with uninfected (uRBC) or infected red blood cells (iRBC) for 6 h in the presence of FCS or FCS supplemented 1% (v/v) autologous serum. **(C,D)** control cultures were incubated in medium alone or with Raji B cells in the presence of Rituximab or isotype control reagent. Bars represent median values and symbols represent individual data points. Paired comparisons between culture conditions were made using Wilcoxon signed rank test. *p < 0.05, **p < 0.01, ***p < 0.001, and ****p < 0.0001.

The ability of NK cells from Gambian and UK adults to mount responses to *Plasmodium falciparum* infected erythrocytes (iRBC) was tested within PBMC after 6 h of culture in the presence or absence of autologous plasma. We observed low frequency but significant induction of IFN-γ in NK cells from Gambian donors in response to iRBC compared to uninfected erythrocytes (uRBC) and these responses were further enhanced in the presence of autologous plasma (Figure 1A). Only occasional responses to iRBC were observed in malaria-unexposed UK individuals either with or without autologous plasma. Similar patterns of responses were observed for degranulation with Gambians, again, having the highest frequencies of CD107a+ cells in the presence of autologous plasma (Figure 1B). By contrast, NK cells from UK and Gambian donors had a similar capacity to produce IFN-γ and to degranulate in response to the control stimulus, Raji B cell tumor + Rituximab (Figures 1C,D).

### Malaria Immune Serum Promotes Antibody-Mediated Responses in Both Gambian and UK Adults

**Figure 2:**
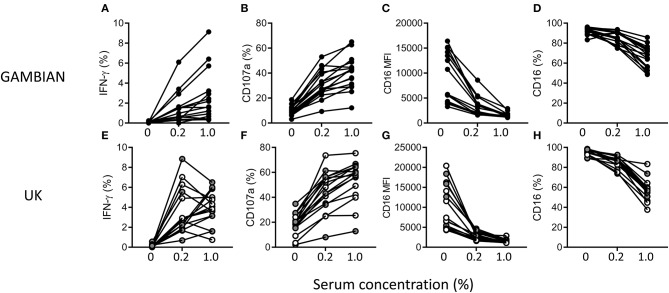
Antibody-dependent NK cell responses in the presence of malaria-immune standard serum. Frequencies of gated CD56dim NK cells expressing **(A,E)** IFN-γ and **(B,F)** CD107a **(C,G)** CD16 and **(D,H)** the MFI for CD16 after 6 h of culture with iRBC in the absence or presence of 0.2% or 1% (v/v) standard malaria-immune serum. Responses are shown for Gambian (**A–D**, *n* = 16) or UK (**E–H**, *n* = 16) individuals. UK HCMV– and HCMV+ individuals are indicated by open and gray symbols, respectively.

To assess the impact of NK cell differentiation and adaptation on antimalarial NK cell responses, responses to iRBC were compared between NK cells from Gambian and UK adults in the presence of a standard anti-malarial serum (Figure 2). NK cells from both Gambian (Figures 2A–D) and UK (Figures 2E–H) individuals mounted robust IFN-γ and CD107a responses, with reciprocal reduction in the frequencies and MFI for CD16 (FcγRIII) expression, in hyper-immune serum (Figures 2A–H).

### Enrichment of Highly Differentiated Adaptive NK Cells in Gambian Adults

**Figure 3:**
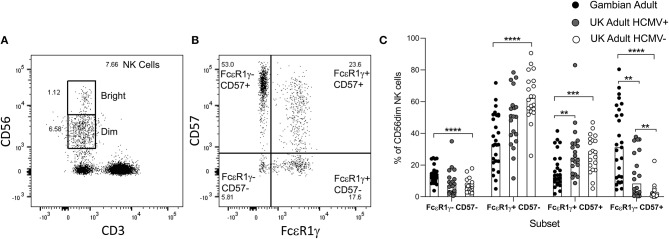
Distribution of canonical and adaptive NK cell subsets. **(A,B)** Gating strategy for NK cell subsets based on co-expression of FcεR1γ and CD57. **(C)** Frequencies of canonical and adaptive NK cell subsets within CD56dim NK cells were determined in malaria- exposed Gambian (*n* = 26) and HCMV+ (*n* = 15) and HCMV– (*n* = 17) malaria-unexposed UK adults. Bars represent median values and symbols represent individual data points. Comparisons between groups were made using Mann-Whitney u test. **p < 0.01, ***p < 0.001, ****p < 0.0001.

We next examined the frequencies of adaptive FcεR1γ- NK cell subsets and the expression of the differentiation marker CD57 in 26 heathy Gambian adults gated as shown in Figures S1A–D (Figures 3A,B) and compared these to the frequencies in 15 HCMV+ and 17 HCMV– UK adults (Figure 3C). Gambian adults had the highest frequencies of highly differentiated, adaptive FcεR1γ-CD57+ NK cells. HCMV-infected UK adults had lower overall frequencies of adaptive cells and these cells were observed only occasionally in HCMV– uninfected individuals (Figure 3C). Reciprocally, higher frequencies of FcεR1γ+CD57– were observed in UK individuals (especially among HCMV-uninfected UK donors) with similar frequencies of FcεR1γ-CD57– and FcεR1γ+CD57+ subsets being observed in all groups (Figure 3C). We observed no difference in the resting frequencies of CD56dim NK cells between Gambian, UK HCMV+ and UK HCMV– individuals, indicating that the differences in the proportions of canonical and adaptive NK cells reflect redistributions within the CD56dim compartment (Figure S1E).

### Adaptive NK Cells Dominate the Antibody-Dependent Responses of Gambian Individuals

**Figure 4:**
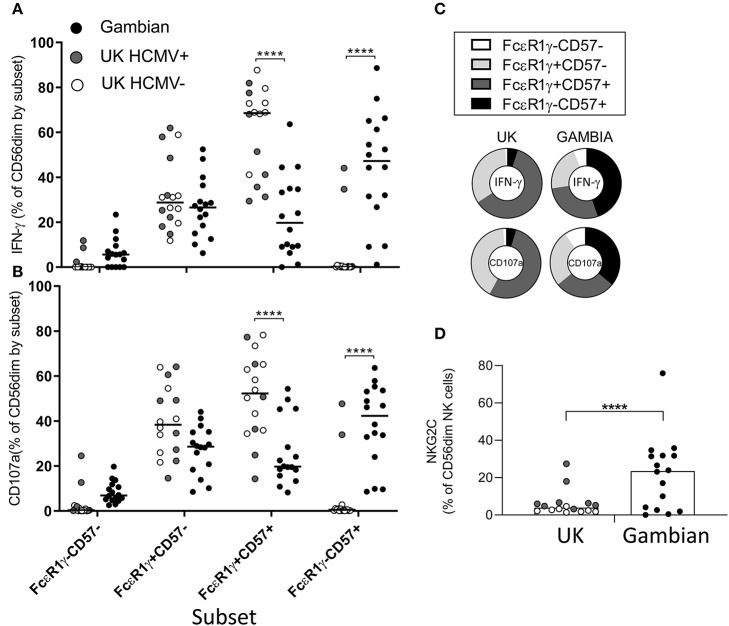
Preferential responsiveness of adaptive NK cells in Gambian individuals. Distribution of **(A)** IFN-γ and **(B)** CD107a expression within back-gated NK cell subsets from UK (*n* = 16) and Gambian individuals (*n* = 16, closed circles) after stimulation with iRBC in the presence of 1% pooled standard malaria immune standard serum. **(C)** Distribution charts showing enrichment of IFN-γ and CD107a responses within FcεR1γ+CD57+ cells of UK individuals and within FcεR1γ-CD57+ adaptive NK cells of Gambian individuals. **(D)** Frequencies of NKG2C+ NK cells in UK and Gambian individuals. **(A,B,D)** Bars represent median values and symbols represent individual data points. HCMV− and HCMV+ UK individuals are represented by open and gray symbols. Comparisons between groups were made using Mann-Whitney u test. ****p < 0.0001.

To assess the impact of differing subset distribution, we back-gated on the IFN-γ+ and CD107a+ NK cell populations to determine the frequencies of each NK cell subset among the iRBC-responding population (Figure 4). For Gambian adults, the highest frequencies of IFN-γ producing cells were found within the highly differentiated, FcεR1γ-CD57+ subsets, whereas for UK individuals IFN-γ was observed predominantly within canonical FcεR1γ+CD57+ cells (Figure 4A). Similarly, degranulation responses occurred predominantly within FcεR1γ-CD57+ cells in Gambian donors but among the FcεR1γ+CD57+ subset in UK donors (Figure 4B). We saw no overall difference in responses between HCMV+ and HCMV– UK individuals. The overall distribution of responses for each group are shown in Figure 4C. However, in two HCMV+ UK individuals with expansions of adaptive NK cells, the NK cell response to iRBC was similar (in subset distribution) to that of the Gambian donors (Figures 4A,B). These two individuals had similar *ex vivo* frequencies of NKG2C+ NK cells to many of the Gambian donors, although the presence of NKG2C expansions did not appear to influence the distribution of FcεR1γ/CD57 defined responses of Gambian donors (Figure 4D). These data are consistent with a functional adaptation of HCMV-associated NK cell expansions toward antibody dependent responses.

### IL-18 Independence of Adaptive NK Cell Responses to iRBC

**Figure 5:**
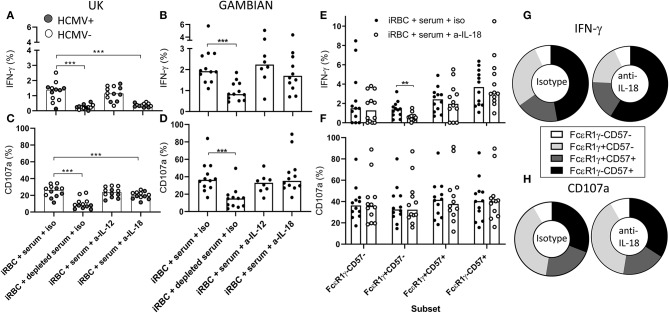
Antibody-mediated adaptive NK cell responses to iRBC are IL-18 independent. Responses of UK (*n* = 12, **A,B**) or Gambian individuals (*n* = 12, **C,D**) to iRBC in the presence of 1% pooled standard malaria immune serum or the same serum depleted of IgG. Responses of HCMV+ UK individuals are represented by gray symbols. Cultures were treated either with isotype control reagent or anti-IL-12 or anti-IL-18 neutralizing antibodies and **(A,C)** IFN-γ or **(B,D)** CD107a measured after 6 h of culture. **(E,F)** frequencies of **(E)** IFN-γ and **(F)** CD107a production within canonical or adaptive NK cell subsets measured after incubation with immune serum in the presence of anti-IL-18 neutralizing or isotype control reagents. **(G,H)** back-gating of **(G)** IFN-γ producing cells or **(H)** CD107a expressing cells showing the relative contributions of FcεR1γ/CD57 defined subsets to these responses. Bars represent median values and symbols represent individual data points. Paired comparisons between conditions were made using Wilcoxon signed rank test. **p < 0.01, ***p < 0.001.

The expression of key elements of cytokine signaling pathways, particularly for IL-12 and IL-18, is lost in adaptive NK cells. We therefore tested whether antibody-dependent responses of iRBC were additionally supported by accessory cytokines. We cultured PBMC from Gambian or UK subjects in the presence of malaria immune serum and with neutralizing antibodies against IL-12, IL-18, or isotype control antibodies (Figure 5). We observed that, in UK individuals, IL-18 blockade reduced antibody-dependent IFN-γ production to the background frequencies observed in IgG-depleted serum (Figure 5A) and resulted in a partial inhibition of degranulation (Figure 5C). Interestingly, we observed no effect of IL-12 blockade on these responses (Figures 5A,C) despite this cytokine having been previously implicated in antibody-independent responses to *P. falciparum* in UK individuals (22, 23). In contrast, blockade of neither IL-18 nor IL-12 significantly reduced the frequencies of IFN-γ or CD107a expressing total CD56dim NK cells responding to iRBC in the presence of malaria immune serum in Gambian subjects (Figures 5B,D). To further establish whether NK cell subsets differ in their reliance on IL-18 for antibody-dependent induction of IFN-γ production or CD107a expression by iRBC, we examined the subset distribution of these responses in Gambian individuals with or without IL-18 neutralization (Figures 5E,F). Interestingly, IL-18 neutralization significantly reduced frequencies of IFN-γ producing cells only within the less differentiated, canonical, FcεR1γ+CD57– NK cell subset (Figure 5E) and there was no significant impact of IL-18 neutralization on the CD107a response in any NK cell subset (Figure 5F). Back-gating on these responses revealed an overall increase in representation of FcεR1γ-CD57+ NK cells within the NK cell IFN-γ in the presence of IL-18 neutralizing antibody but no impact on the CD107a response (Figures 5G,H).

### Adaptive NK Cells Dominate the Antibody-Dependent Responses of Purified Cells

**Figure 6:**
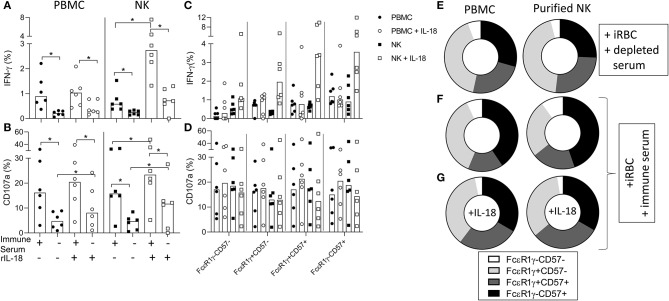
Antibody-dependent responses of purified NK cells are enriched within adaptive NK cells. **(A,B)** Frequencies of CD56dim NK cells within PBMC or purified NK cells producing **(A)** IFN-γ or **(B)** CD107a in response to iRBC in the presence of 1% pooled standard malaria immune serum or antibody depleted serum and the effect of exogenous IL-18. **(C,D)** Frequencies of **(C)** IFN-γ+ and **(D)** CD107a+ NK cells responding within gated FcεR1γ/CD57 defined NK cell subsets. **(E)** Overall subset distributions within total CD56dim NK cells cultured in the presence of antibody depleted serum. **(F,G)** FcεR1γ/CD57 defined subset distributions for responses of PBMC or purified NK cells within back-gated IFN-γ producing cells: **(F)** iRBC + immune serum, **(G)** iRBC + immune serum + 10 ng/ml rIL-18. Data are shown for matched PBMC and enriched NK cells from 6 Gambian individuals. Bars represent median values and symbols represent individual data points. Paired comparisons between culture conditions were made using Wilcoxon Signed rank test. *p < 0.05.

We then examined the ability of purified NK cells from Gambian individuals to mount antibody-dependent responses to iRBC (Figure 6). Significant antibody-dependent IFN-γ and degranulation responses were observed within purified NK cells, indicating that these responses could occur independently of accessory cell-derived factors (Figures 6A,B). Addition of recombinant IL-18 revealed significant synergy between IL-18 and malaria immune serum for IFN-γ production and degranulation responses whereas only limited additive effects were observed in the presence of antibody depleted serum (Figures 6A,B). IFN-γ responses of PBMC or purified NK cells to iRBC + immune serum were enriched within gated FcεR1γ-CD57+ NK cells compared to canonical NK cell subsets whereas degranulation responses were more evenly distributed across all FcεR1γ/CD57 defined CD56dim NK cells subsets (Figures 6C,D). Interestingly, however, the addition of exogenous IL-18 to PBMC or purified NK cells resulted in increased frequencies of antibody-dependent IFN-γ producing NK cells within all FcεR1γ/CD57 defined subsets, indicating that adaptive NK cell responses could be augmented by relatively high physiological concentrations of this cytokine (Figure 6C). Relative to the overall distribution within total CD56dim NK cells cultured with antibody depleted serum (Figure 6E), back-gating of IFN-γ responses in malaria immune serum confirmed an over-representation of FcεRγ-CD57+ NK cells (Figure 6F). Interestingly, despite clear synergy with anti-malarial antibody, the relative contribution of adaptive FcεRγ-CD57+ NK cells to the overall IFN-γ response was reduced in the presence of exogenous IL-18 (Figure 6G). These data are consistent with accessory cell-independence for antibody-dependent responses of adaptive NK cell responses to iRBC. An even subset distribution was observed for degranulation responses in the presence or absence of exogenous IL-18 (Figure S2).

### IL-18Rα Distribution on NK Cell Subsets

**Figure 7:**
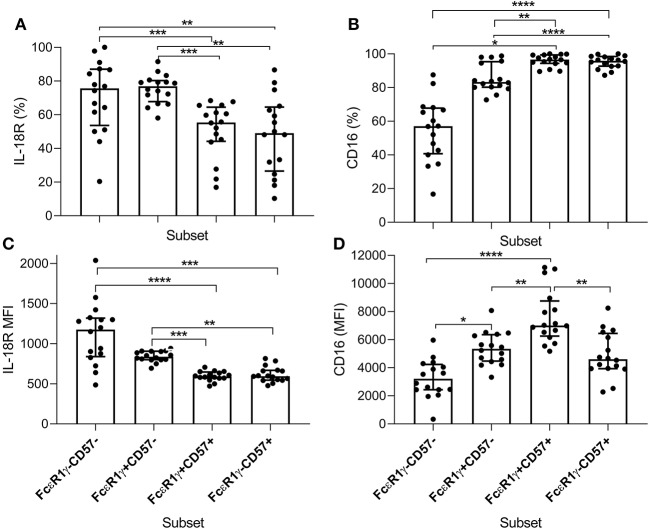
Distribution of IL-18Rα and CD16 within NK cell subsets of Gambian individuals. Frequencies of **(A)** IL-18Rα and **(B)** CD16 expressing subsets were measured *ex vivo* in 16 Gambian adults and are shown within gated CD56dim NK cells alongside **(C,D)** the corresponding MFI for these receptors. Bars represent median values and symbols represent individual data points. Unbiased paired comparisons between subsets were made using Freidman's test with Dunn's correction for multiple comparisons. *p < 0.05, **p < 0.01, ***p < 0.001, and ****p < 0.0001.

To examine further the basis for IL-18 independence of adaptive NK cells for antibody- dependent responses to *P. falciparum*, we examined expression of IL-18Rα and CD16 within FcεR1γ/CD57-defined NK cell subsets (Figure 7). Consistent with a lack of IL-18 involvement in antibody-dependent responses of adaptive NK cells to *P. falciparum*, we observed the lowest frequencies of IL-18Rα expressing cells within adaptive FcεR1γ-CD57+ NK cells (Figure 7A). The lowest MFI for IL-18Rα expression was observed in highly differentiated canonical and adaptive NK cell subsets (Figure 7C). In Gambians, a gradated increase in the frequency of CD16 expression on CD56dim NK cells was observed with FcεR1γ+CD57+ and FcεR1γ-CD57+ NK cells having the highest proportion (Figure 7B). Interestingly the MFI for CD16 was highest in canonical CD57+ NK cells (Figure 7D), indicating that, although these cells were examined *ex vivo*, CD16 may be more susceptible to downregulation on adaptive NK cells in our study population.

Overall, these data support an important role for adaptive NK cells in mounting antibody-dependent responses to *P. falciparum* iRBC erythrocytes in a manner that is independent of accessory cells and accessory-cell derived cytokines. These findings therefore support a contribution of adaptive NK cells to anti-malarial immunity which limits reliance on inflammatory pathways.

## Discussion

Effective anti-malarial immunity is characterized by the ability to prevent parasite multiplication and to kill and remove parasites and iRBC whilst at the same time minimizing inflammation and the consequent febrile response; this is largely attributed to the gradual acquisition of populations of regulatory cells that suppress inflammation (34) and to a switch from predominately cell-mediated immune effector mechanisms to antibody-mediated parasite clearance. However, given the largely intracellular nature of the parasite lifecycle, synergy between antibodies and cytotoxic/phagocytic cells is more likely to lead to efficient clearance of infected cells than purely antibody-mediated mechanisms such as neutralization or complement fixation. The conundrum, therefore, is how to ensure effective co-operation between antibodies and effector cells without the need for inflammatory cytokines to activate cytotoxicity and/or phagocytosis. This study begins to answer this conundrum by demonstrating the impact of human NK cell differentiation and adaptation on responses to malaria parasites, enabling NK cell activation by antibody without any need for cytokines. This study also begins to inform the ongoing debate around differences in immune responses between individuals living in “westernized” environments and those living in low and middle income countries and the consequences for vaccine efficacy (35–39). Specifically, this study shows that African individuals possess significantly higher frequencies of highly differentiated, adaptive (FcεR1γ-) NK cells than either HCMV-infected or -uninfected UK individuals of a similar age and that these differences in the distribution of NK cell subsets impacts on their activation by *P. falciparum*-infected erythrocytes, in particular their ability to be activated by antigen-antibody complexes in the absence of inflammatory cytokines. Our data are thus in line with recent studies showing that purified NK cells can inhibit the growth of *P. falciparum* in culture in a manner that is dependent upon malaria-specific antibodies but independent of accessory cells (29).

We have shown that malaria hyper-immune serum, but not IgG-depleted immune serum, supported strong cytokine and degranulation responses to iRBC by NK cells of both malaria-exposed Gambians and malaria-naïve UK individuals, confirming the importance of malaria specific antibody. Using autologous plasma to support the antibody-dependent responses to iRBC, the level of antibody to antigens expressed on the erythrocyte surface is likely to contribute to differences in magnitude of the NK cell response; clonally variant antigens including PfEMP-1 and Rifins have been shown to contribute to the antibody-dependent NK cell response (30). All of the adult Gambians selected for this study were seropositive for the merozoite-expressed apical membrane antigen-1 (AMA-1), as determined by ELISA. We observed no direct relationship between the levels of anti-AMA-1 antibodies (or a further 17 conserved merozoite-expressed antigens) and the magnitude of the NK cell response, consistent with the notion that crosslinking of merozoite antigens does not make a major contribution to NK activation by intact, parasitized RBCs (data not shown).

NK cells from both Gambian and UK donors mount robust antibody-dependent responses to malaria-infected erythrocytes in the presence of a standard malaria hyper-immune sera. However, different NK cell subsets dominate these responses depending on the donor origin. In UK donors, differentiated canonical FcεR1γ+CD57+ cells dominate antibody-dependent responses, whereas in Gambian donors these responses occur preferentially within adaptive FcεR1γ-CD57+ NK cells. Less differentiated CD57–CD56dim– NK cells were the principal subsets reliant on IL-18 for antibody-dependent responses in both study populations. This is consistent with the previously reported predominance of less differentiated CD56bright or CD56dim CD57– NK cells in responses to cytokines (in the absence of CD16 crosslinking) (2, 3, 31, 40). Remarkably, and distinct from previous findings in murine *P. chabaudi* malaria (41) early, antibody-dependent IFN-γ responses to *P. falciparum* iRBC were entirely independent of IL-12 (and IFN-αβR signaling, data not shown) within all NK cell subsets. Previous studies have reported that IL-18, in particular, synergises with antibody Fc receptor crosslinking for the promotion of enhanced antibody-mediated NK cell responses (3, 42): low concentrations of IL-18 synergised with CD16 cross-linking or antibody-mediated responses to inactivated H3N2 influenza virus particles for enhanced IFN-γ, CD107a and CD25 expression (3, 42). Whilst synergy between CD16-mediated and IL-18-mediated signals promotes potent early NK cell responses (within 6 h of *in vitro* culture), we cannot exclude that the response kinetic differs for distinct adaptive or canonical NK cell subsets (3). However, our data indicate that potent NK cell IFN-γ and CD107a responses, with reciprocal down regulation of CD16 expression. occur in both UK and Gambian individuals within 6 h of culture with iRBC and malaria hyper-immune serum. Synergy between CD16 and endogenous IL-18 may therefore be required to promote robust early responses in canonical NK cells, but this may not required for the optimal response of adaptive cells. Equally importantly, addition of exogenous recombinant IL-18 to PBMC or purified NK cell cultures indicates that higher physiological concentrations of IL-18 may indeed influence the activation of adaptive NK cells. CD16 associates with disulphide-linked homodimers or heterodimers of the TCRζ and/or the FcRγ chains and signaling is mediated via Src-family kinase-mediated tyrosine phosphorylation ultimately leading to PI3K, Vav1, PLC-γ1, and PLC-γ2 activation (43, 44). IL-18 utilizes the TIR domain of MyD88 to access the NFκB signaling pathway (45, 46). Importantly, our data indicate a significant role for endogenous IL-18 on antibody mediated IFN-γ responses with only limited effects of IL-18 neutralization on degranulation responses. It is therefore likely that crosstalk between these pathways is responsible of the enhancement of antibody mediated effects on cytokine production.

Consistent with previous studies (2), IL-18Rα expression in NK cells from Gambian individuals correlated with the extent of NK cell differentiation. IL-18Rα+ NK cells are less frequent (and the MFI of receptor expression is lower) in more differentiated CD57+ NK cells, consistent with their dependence on this cytokine for optimal antibody induced IFN-γ production to iRBC but less so for degranulation. It has been reported that targeting of HLA-E transfected cell lines by CD56dim CD57+NKG2C+ NK cells, in the absence of antibody-mediated signals, was enhanced by IL-18, implying that “adaptive” NK cells defined by these receptors maintain some components of the IL-18R signaling pathway (47); these studies also reported maintenance of IL-18RAP transcript expression in NKG2C+ NK cells, albeit with reduced levels of NFκB phosphorylation (47). However, in these studies “adaptive” NKG2C+ NK cells were not classified according to FcεR1γ expression. In our study NKG2C expressing NK cells are significantly more prevalent in Gambian than in UK individuals and are equally represented within the FCεR1γ+CD57+ canonical and FcεR1γ-CD57+ adaptive subset suggesting that CD56dim CD57+NKG2C+ NK cells comprise both IL-18-dependent (FcεR1γ+) and IL-18-independent (FcεR1γ-) subpopulations (Figure S3). We also observed higher frequencies of CD2 expression within FcεR1γ-CD57+ NK cells with the potential for LFA-3 mediated interactions (Figure S3).

Whilst NK cell responses to iRBC vary between individuals and between populations, this is only partially explained by differences in the proportions of NK cells in particular NK cell subsets. Even in controlled conditions, using standardized parasite preparations and a standard hyper-immune serum, and after accounting for differences in proportions of NK cell subsets, there was considerable variation between individuals in their response to iRBC. It is therefore likely that the relationship between malaria-specific antibody and the extent of NK cell activation is a complex one, which goes beyond the magnitude of the antibody response and the intrinsic capacity of distinct NK cell differentiation subsets to respond. Variation in the frequencies of CD16+ NK cells and the extent of CD16 expression within canonical and adaptive NK cell subsets (see Figure 7) are likely to play a role in determining the magnitude of antibody-dependent responses. Whilst the vast majority of FcεR1γ-CD57+ adaptive NK cells are CD16+, the MFI for CD16 expression was lower in this subset than in the canonical FcεR1γ+ subset; this may reflect more frequent cross-linking by immune complexes and subsequent metalloprotease-mediated down-regulation of CD16 within adaptive NK cells in our Gambian study subjects, consistent with previous reports (48, 49). Additionally, and consistent with previous estimates in African populations (50, 51), the frequency of the well-documented high affinity 158-V allele of CD16 is relatively high in our Gambian study population and likely contributes to variation in both expression of CD16 and the magnitude of the antibody-dependent NK cell response. Finally, individual differences in the frequency and MFI of IL-18Rα expression on NK cells, or frequencies of IL-18 producing accessory cells or the overall concentration of IL-18, could also contribute to variation in antibody-dependent responses, especially in the less differentiated subsets.

In summary, there is accumulating evidence that NK cells may play an important role in antibody-mediated control of malaria infections and may be pivotal in ensuring clearance of iRBC in situations where potentially damaging inflammatory responses are down regulated. The quality of the antibody response, in combination with the maturation status of the NK cell population (which in itself is affected by prior infection history) and the immune regulatory environment of the host will all play a role in determining the effectiveness of these ADCC mechanisms. This study is limited in that it does not indicate whether NK cell-mediated ADCC or ADCI responses contribute directly to anti-malarial immunity and future field studies should compare *Plasmodium* specific antibody responses, inflammatory cytokine measures, adaptive NK cell frequencies and NK cell ADCC/ADCI responses in malaria immune and non-immune individuals in an endemic setting. Further studies should also address the nature of the antibodies and the NK cells which most efficiently trigger NK cell-mediated killing of *P. falciparum* iRBCs, and the impact of the cytokine environment (both inflammatory and anti-inflammatory). These factors may also be important in the context of malaria vaccines, where the choice of adjuvant could influence the cytokine-induced pre-activation of canonical NK cell subsets and population differences in the frequencies of adaptive NK cells could impact their role as effectors in the vaccine induced response. The impact of genetic variation—diversity in HLA and killer cell immunoglobulin-like receptors (52–54) as well as polymorphisms in other NK cell receptors such as CD16 and NKG2C—on the diversity of NK cell responses within and between populations should also be considered.

## Data Availability Statement

All datasets generated for this study are included in the article/Supplementary Material.

## Ethics Statement

The studies involving human participants were reviewed and approved by the London School of Hygiene and Tropical Medicine Research Ethics Committee and the Gambia Government/MRC Gambia Joint Ethics Committee. The patients/participants provided their written informed consent to participate in this study.

## Author Contributions

SS designed and performed experiments, analyzed data, and wrote the manuscript. AP and DB contributed to laboratory work, directed parasitological aspects of the work, and edited the manuscript. ER directed research and wrote the manuscript. MG directed research, designed, performed experiments, analyzed and interpreted data, and wrote the manuscript. All authors contributed to manuscript revision, read, and approved the submitted version.

### Conflict of Interest

The authors declare that the research was conducted in the absence of any commercial or financial relationships that could be construed as a potential conflict of interest.

## Supplementary Material

Figure S1 Gating strategy and comparative frequencies of CD56dim NK cells. **(A)** NK cell gate **(B)** CD56dim cell gate. **(C)** IFN-γ production and **(D)** CD107a expression gates are shown. Data are shown for IFN-γ and CD107a responses to iRBC in the presence or absence of malaria hyper immune serum at the indicated concentrations **(C,D)**. **(E)** Frequencies of gated CD56dim NK cells in Gambians compared to UK HCMV+ and UK HCMV– individuals. Bars represent median values and symbols represent individual data points.

Figure S2 Impact of exogenous IL-18 on the subset distribution for CD107a expression frequencies within PBMC and purified NK cells. Charts show the distribution of FCεR1γ/CD57 defined subsets within gated CD107a+CD56dim NK cells within PBMC or NK cells in the absence **(A)** or presence of exogenous IL-18 **(B)**. Combined distributions are shown for 6 individuals.

Figure S3 *Ex vivo* distribution of CD2 and NKG2C within FCεR1γ/CD57 defined NK cell subsets. **(A)** NKG2C and **(B)** CD2 expression was monitored in gated NK cell subsets in Gambian individuals (NKG2C, *n* = 16; CD2, *n* = 26). Bars represent median values and symbols represent individual data points. Unbiased paired comparisons between subsets were made using Freidman's test with Dunn's correction for multiple comparisons. ***p* < 0.01, ****p* < 0.001 and *****p* < 0.0001.

## Abbreviations

NK: natural killer
*P. falciparum*: *Plasmodium falciparum*
uRBC: uninfected red blood cells
iRBC, infected red blood cells, IL-18: Interleukin 18
ADCC: antibody dependent cellular cytotoxicity.
