# Supplementary figures and images for: Differential IL-18 Dependence of Canonical and Adaptive NK Cells for Antibody Dependent Responses to P. falciparum
Source: Front Immunol. 2020 Mar 31;11:533. doi: 10.3389/fimmu.2020.00533 (PMC7137096; doi:10.3389/fimmu.2020.00533)

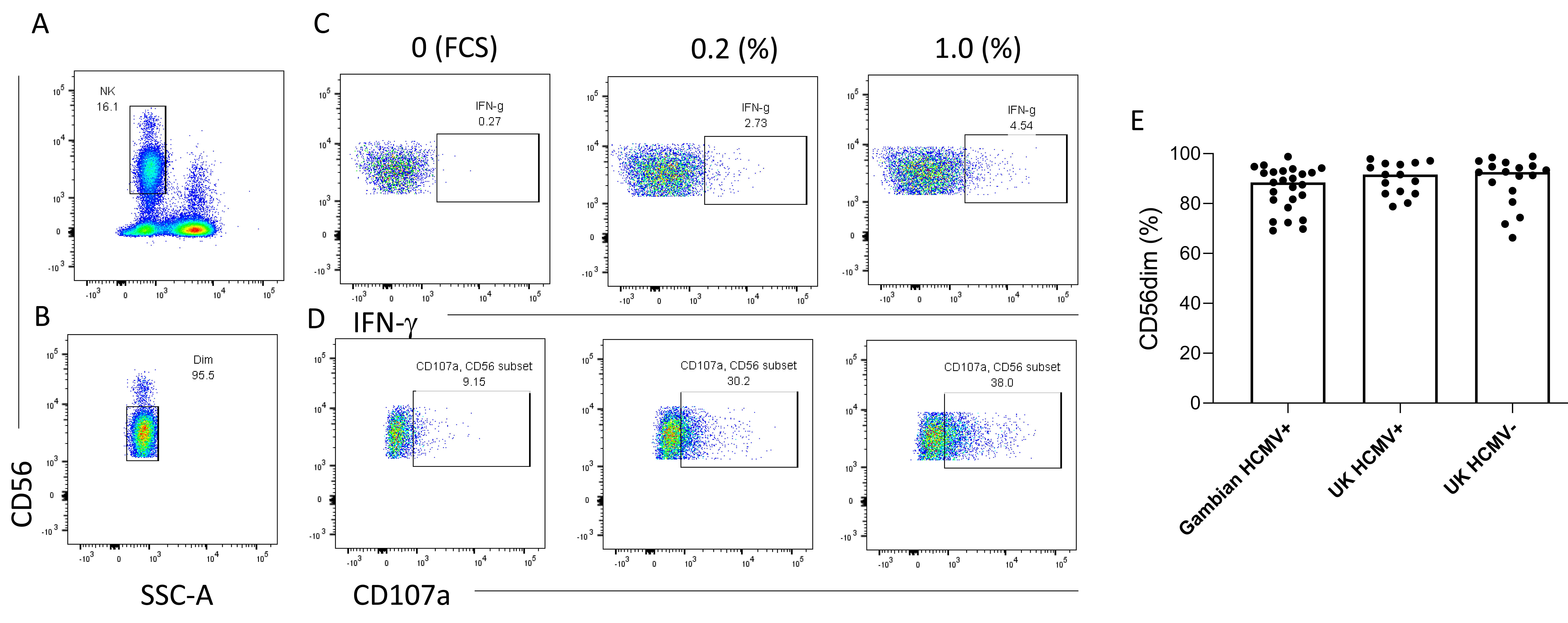

Supplement: Figure S1 — Gating strategy and comparative frequencies of CD56dim NK cells. (A) NK cell gate (B) CD56dim cell gate. (C) IFN-γ production and (D) CD107a expression gates are shown. Data are shown for IFN-γ and CD107a responses to iRBC in the presence or absence of malaria hyper immune serum at the indicated concentrations (C,D). (E) Frequencies of gated CD56dim NK cells in Gambians compared to UK HCMV+ and UK HCMV– individuals. Bars represent median values and symbols represent individual data points. [file Image_1.TIF]

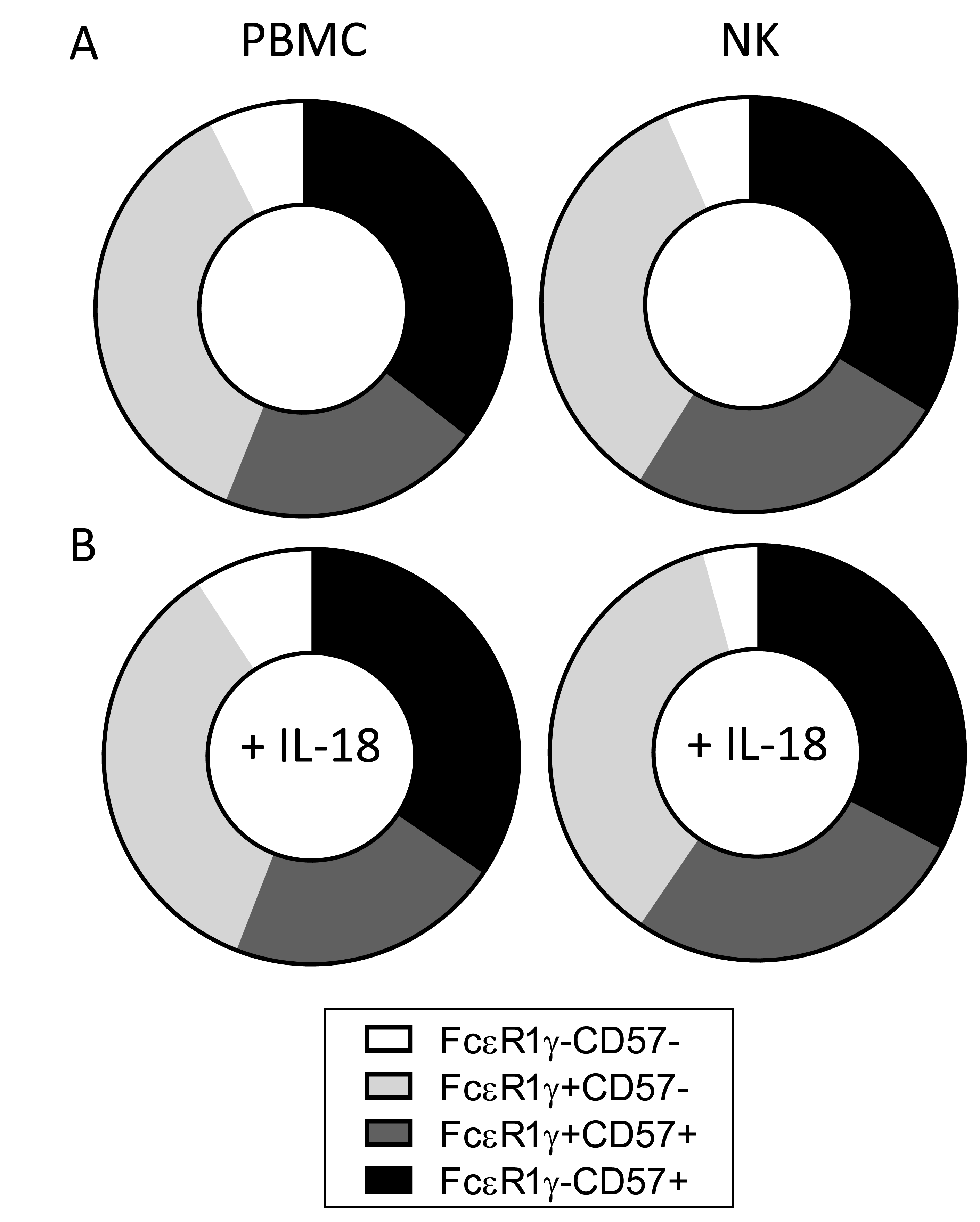

Supplement: Figure S2 — Impact of exogenous IL-18 on the subset distribution for CD107a expression frequencies within PBMC and purified NK cells. Charts show the distribution of FCεR1γ/CD57 defined subsets within gated CD107a+CD56dim NK cells within PBMC or NK cells in the absence (A) or presence of exogenous IL-18 (B). Combined distributions are shown for 6 individuals. [file Image_2.TIF]

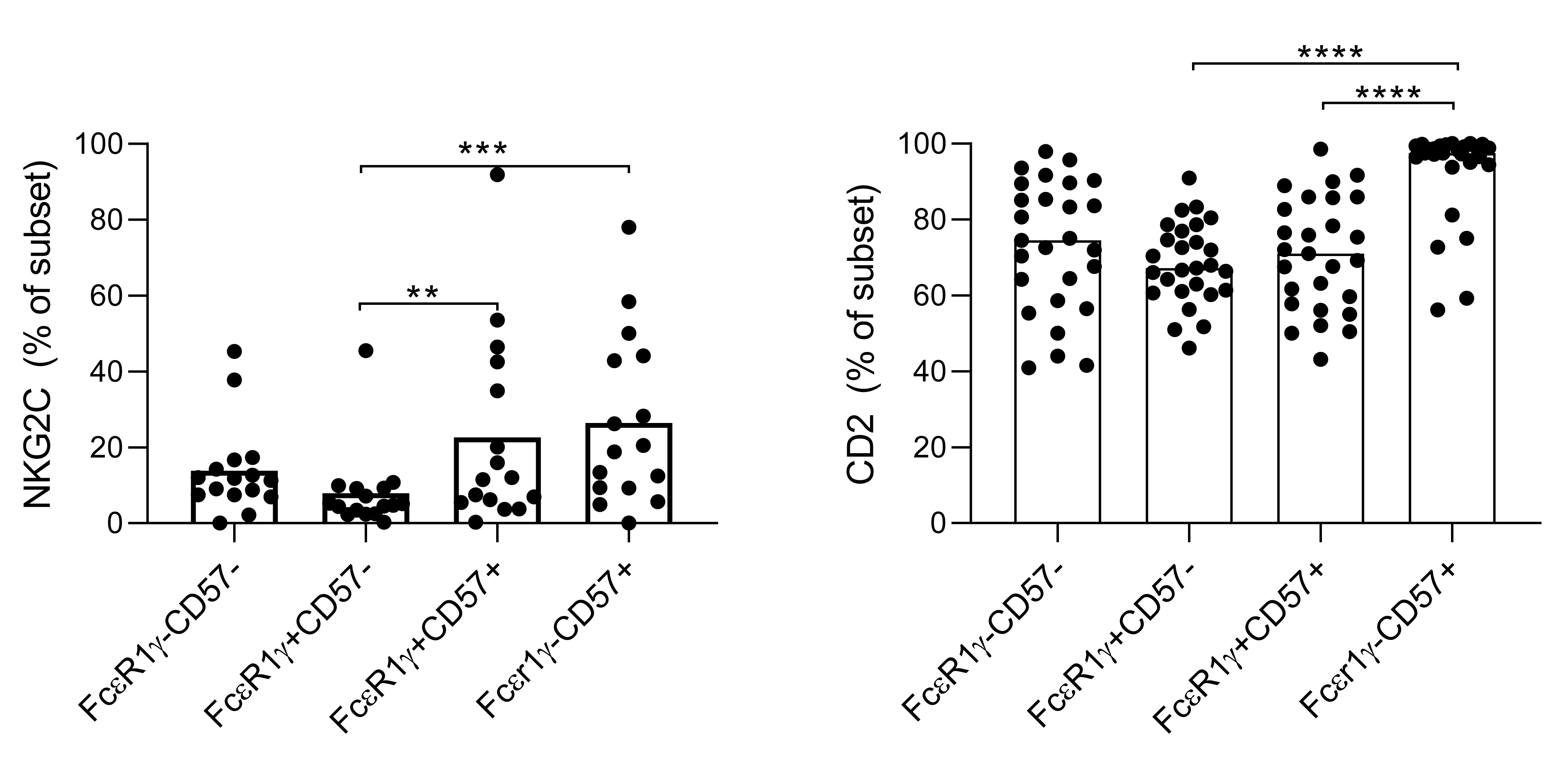

Supplement: Figure S3 — Ex vivo distribution of CD2 and NKG2C within FCεR1γ/CD57 defined NK cell subsets. (A) NKG2C and (B) CD2 expression was monitored in gated NK cell subsets in Gambian individuals (NKG2C, n = 16; CD2, n = 26). Bars represent median values and symbols represent individual data points. Unbiased paired comparisons between subsets were made using Freidman's test with Dunn's correction for multiple comparisons. **p < 0.01, ***p < 0.001 and ****p < 0.0001. [file Image_3.TIF]
